# Adductor Muscle Contraction Under Deep Neuromuscular Blockade During TURBT Under General Anesthesia: Is Obturator Nerve Block Still Necessary?—A Prospective, Single-Arm, Exploratory Study

**DOI:** 10.3390/medicina61071207

**Published:** 2025-07-01

**Authors:** Su Yeon Cho, Ki Tae Jung

**Affiliations:** 1 Department of Anesthesiology and Pain Medicine, Chosun University Hospital, Gwangju 61453, Republic of Korea; isycho@chosun.ac.kr; 2 Department of Anesthesiology and Pain Medicine, College of Medicine and Medical School, Chosun University, Gwangju 61452, Republic of Korea

**Keywords:** adductor muscles, general anesthesia, neuromuscular blockade, nerve block, obturator nerve, urinary bladder neoplasms/surgery

## Abstract

*Background and Objectives*: Obturator reflex during transurethral resection of bladder tumors (TURBT) can cause serious complications, such as bladder perforation, hemorrhage, and incomplete resection. Although obturator nerve block (ONB) is routinely recommended under spinal anesthesia, it is often omitted under general anesthesia (GA) based on the assumption that neuromuscular blockade (NMB) alone prevents adductor muscle contractions. However, clinical observations suggest that the obturator reflex may still occur under deep NMB. This study aimed to determine whether adductor longus muscle (ALM) contraction persists under GA with deep NMB during TURBT. *Materials and Methods*: Thirty patients scheduled for TURBT under GA were prospectively enrolled. A selective ONB was performed under ultrasound and nerve stimulator guidance. After establishing the baseline current intensity for ALM contraction, neuromuscular monitoring was initiated, and rocuronium (0.6 mg/kg) was administered. Stimulation thresholds required to induce ALM contraction were sequentially assessed at decreasing Train-of-Four ratio (TOFr) stages (90% to 10%) and Train-of-Four count (TOFc) stages (3 to 0). Final measurements were repeated 1 min after achieving TOFc 0. Changes in stimulation intensity were analyzed using a linear mixed-effects model (LMM). *Results*: As NMB deepened, the current intensity required to provoke ALM contraction progressively increased: 0.51 ± 0.25 mA at TOFr 90%, 1.66 ± 0.53 mA at TOFr 10%, 2.04 ± 0.66 mA at TOFc 0, and 2.61 ± 0.29 mA at 1 min after TOFc 0. Notably, all patients demonstrated ALM contraction at TOFc 0 and thereafter, confirming the persistence of the obturator reflex despite complete NMB. LMM analysis revealed a significant trend of increasing stimulation thresholds with progressive NMB depth (β = 0.133, *p* < 0.001). *Conclusions*: Adductor muscle contractions in response to obturator nerve stimulation persist even under deep NMB. These findings raise concerns that deep NMB alone may be insufficient to prevent obturator reflex and suggest that ONB should be considered as an adjunctive practice during TURBT under GA in patients at risk.

## 1. Introduction

Transurethral resection of bladder tumors (TURBT) is widely performed as a first-line treatment for non-muscle invasive bladder cancer [1]. However, tumors located at the inferolateral wall of the bladder pose a unique challenge due to the proximity of the obturator nerve [2]. As the obturator nerve travels close to the inferolateral bladder wall, resecting a tumor on the lateral bladder wall can directly stimulate the nerve [3]. Stimulation of this nerve during electrocautery may provoke a sudden contraction of the adductor muscles—known as the obturator reflex—which can result in complications such as bladder wall injury, excessive bleeding, or incomplete resection [2].

Anesthetic management plays a critical role in minimizing these risks. TURBT is often performed under regional anesthesia, particularly spinal anesthesia, which may fail to completely suppress the obturator reflex due to incomplete motor block of the obturator nerve [4]. Moreover, spinal anesthesia is not always feasible in all patients, especially the elderly or those with anatomical or coagulation contraindications. General anesthesia (GA) with neuromuscular blocking agents (NMBAs) is often applied in uncooperative patients, in cases of failed spinal anesthesia, or when adductor spasms persist [3].

To minimize complications caused by the obturator reflex, obturator nerve block (ONB) is frequently performed in conjunction with not only spinal anesthesia but also GA, and it has been shown to reduce the risk of intraoperative adductor contractions effectively [2,3,4,5]. The incidence of the obturator reflex dropped from about 46% to 22% when ONB was used during TURBT [4]. Bladder perforation rates were also markedly decreased in combination with ONB, falling from ~53% (without ONB) to ~11% with an ONB in place [4].

Although ONB is widely regarded as the most effective and reliable method to abolish the obturator reflex, ONB is usually omitted in GA, assuming NMBAs provide sufficient neuromuscular blockade (NMB) and adductor contractions will not occur in deep NMB. However, whether NMB alone adequately suppresses the obturator reflex during TURBT remains controversial, especially when the depth of blockade is suboptimal. Unexpected adductor contractions despite NMB have been reported, possibly due to direct electrical stimulation of the obturator nerve bypassing the neuromuscular junction [6,7].

This paradox—the occurrence of the obturator reflex under deep NMB—raises an important clinical question: Is ONB still necessary in patients undergoing TURBT under GA with deep NMB? In the absence of sufficient evidence, omitting ONB may leave patients vulnerable to the obturator reflex-induced complications during TURBT. To address this issue, the present study investigated whether adductor muscle responses can be evoked during GA with deep NMB and aimed to provide evidence to guide anesthetic planning for reducing the incidence of obturator-related complications during TURBT.

## 2. Materials and Methods

### 2.1. Study Design and Ethical Considerations

This prospective, single-arm, exploratory study was conducted at Chosun University Hospital (Gwangju, Republic of Korea) between June 2023 and January 2024. The study protocol was approved by the Institutional Review Board of Chosun University Hospital (IRB No. CHOSUN 2023-03-031 and approval date of 31 March 2023). This study was registered at ClinicalTrials.gov (Clinical trial registration number: NCT05872451; date: 12 May 2023). All participants provided written informed consent after receiving detailed information about the study protocol and procedures. All study procedures adhered to the principles of the Declaration of Helsinki (2013 revision).

### 2.2. Participants

**Figure 1:**
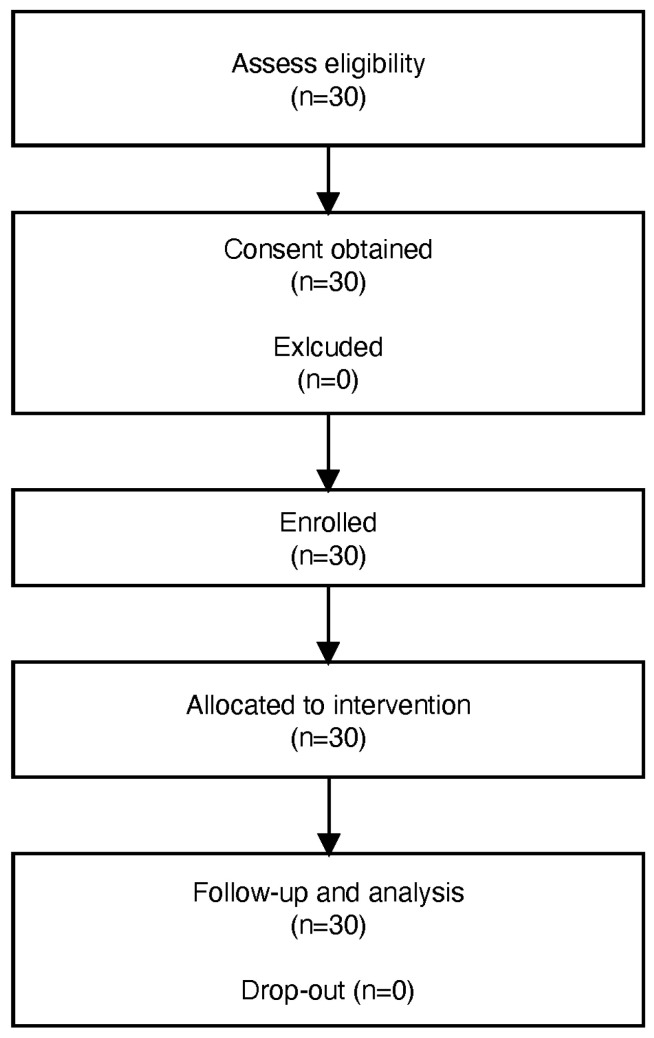
CONSORT flow diagram of participant enrollment and analysis.

A total of 30 patients scheduled for elective TURBT under GA were prospectively enrolled in this study (Figure 1). All patients had bladder tumors located at or near the lateral wall, where the risk of the obturator reflex was considered clinically significant. Enrollment was limited to cases in which the attending urologist specifically requested an ONB due to the tumor’s proximity to the obturator nerve. Eligible participants were adults aged 18 years or older, with American Society of Anesthesiologists physical status I or II, and a body mass index (BMI) between 18.5 and 30.0 kg/m^2^. Patients were excluded if they had a known neuromuscular disorder, a hip joint pathology, an anatomical or functional upper airway abnormality, contraindications to obturator nerve block, such as inguinal lymphadenopathy, perineal infection, or hematoma at the needle insertion site, the inability to tolerate the lithotomy position, or a decline in GA. As this was a single-arm observational study, neither randomization nor blinding was applied during data collection or analysis.

### 2.3. Anesthesia and Neuromuscular Monitoring

All patients were instructed to fast overnight prior to surgery, and no premedication was administered. Upon arrival in the operating room, standard monitoring devices (Carescape monitor, GE Healthcare, Chicago, IL, USA) were applied, including electrocardiography, noninvasive blood pressure measurement, and pulse oximetry. In addition, SedLine^®^ sensors and a SedLine^®^ monitor (Masimo Inc., Irvine, CA, USA) were attached for patient state index (PSI) monitoring.

Following 5 min of preoxygenation via face mask, anesthesia was induced with total intravenous anesthesia (TIVA) using a target-controlled infusion (TCI) system (Perfusor^®^ Space, B. Braun, Melsungen, Germany). Propofol and remifentanil were administered using the Marsh and Minto pharmacokinetic models, respectively. The initial target effect-site concentration (Ce) of propofol was set to 5.0 µg/mL, and that of remifentanil was set to 3.0 ng/mL. Manual mask ventilation was provided during the induction period. No neuromuscular blocking agents (NMBAs) were administered during induction.

When the patient lost consciousness and the PSI value decreased below 50, a supraglottic airway device (i-gel^®^, Intersurgical Ltd., Wokingham, UK) was inserted. The size of the i-gel was selected based on patient weight (size 3 for 30–60 kg, size 4 for 50–90 kg, and size 5 for >90 kg). The correct placement was confirmed by capnography and bilateral chest auscultation. Anesthesia was maintained using TIVA with a 50% mixture of oxygen and air. The Ce of propofol and remifentanil was continuously titrated based on PSI values, targeting a range of 25 to 50, and adjusted further to ensure that hemodynamic parameters remained within 20% of the patient’s baseline. Mechanical ventilation was initially set with a tidal volume of 8 mL/kg and a respiratory rate of 12 breaths per minute. Ventilatory parameters were subsequently modified as needed to maintain an end-tidal CO_2_ between 35 and 40 mmHg and to ensure peak inspiratory pressure remained below 28 mmHg.

Quantitative neuromuscular monitoring was conducted using acceleromyography (TOFscan^®^, IdMed, Marseille, France), in accordance with the consensus guidelines for Good Clinical Research Practice (GCRP) in pharmacodynamic studies of neuromuscular blocking agents [8]. Train-of-Four (TOF) stimulation was applied to the ulnar nerve at the wrist, and evoked responses were recorded at the adductor pollicis muscle on the arm opposite to the one used for noninvasive blood pressure monitoring. Electrical stimuli were delivered every 12 s, using a fixed current of 50 mA and a pulse duration of 0.2 ms, as per device specifications and in line with GCRP recommendations. During the entire monitoring period, the stimulated arm was left uncovered and undisturbed to avoid movement artifacts or external interference with twitch measurements. Neuromuscular monitoring was initiated immediately after establishing the baseline current threshold for adductor muscle contraction via obturator nerve stimulation, as described later in this section.

### 2.4. Obturator Nerve Block and Current Intensity Measurement

With the patient positioned in the lithotomy position, the inguinal region was sterilized using a chlorhexidine-based antiseptic. To eliminate the contribution of the posterior branch of the obturator nerve innervating the adductor longus muscle (ALM), a selective obturator nerve block was performed prior to the study protocol. A high-frequency linear ultrasound probe (Philips Lumify™, Philips Healthcare, Best, The Netherlands) was applied in the transverse orientation to identify the fascial plane between the adductor longus and adductor brevis muscles, which indicates the location of the anterior branch of the obturator nerve. Under real-time ultrasound guidance, a 22-gauge insulated stimulating needle (Stimuplex^®^ D, B. Braun, Melsungen, Germany) was inserted using an in-plane approach. A nerve stimulator (Stimuplex^®^ HNS12, B. Braun, Melsungen, Germany) was connected to the needle for sequential electrical stimulation. Stimulation was initiated at 1.0 mA with a pulse duration of 0.1 ms and a frequency of 2 Hz. The needle position was optimized until ALM contraction occurred at ≤0.5 mA. After negative aspiration, a total of 10 mL of a mixed solution (0.1% lidocaine and 0.5% ropivacaine, 1:1 volume ratio) was slowly injected to block the posterior branch.

After the posterior branch block, the needle was redirected toward the anterior branch of the obturator nerve until visible contraction of the ALM was observed. The current intensity was then slowly decreased to identify the baseline current intensity required to elicit a detectable twitch of the ALM. The needle position was fixed once a consistent response was achieved at low stimulation intensity (typically between 0.3 and 0.5 mA).

Following baseline measurement of current intensity, continuous TOF monitoring was initiated, and 0.6 mg/kg of rocuronium (Esmeron^®^, MSD Ltd., Seoul, Korea) was administered intravenously as a bolus. As NMB progressed, the TOF ratio (TOFr) and TOF count (TOFc) were measured every 12 s. At each stage of neuromuscular depression—defined by decreasing TOFr values (from 90% to 0%) and TOFc levels (from 4 to 0)—electrical stimulation was applied to the obturator nerve through the indwelling stimulating needle. The stimulating current intensity was gradually increased until a visible contraction of ALM was observed. This process was repeated at each TOFr level and TOFc transition, and the current intensity required to elicit adductor muscle contraction was recorded at each stage.

Once complete, NMB was confirmed—defined as a TOFc of 0—the current intensity required to provoke ALM contraction was measured. To further assess the presence of residual obturator reflex under deep NMB, the stimulation was repeated 1 min later, with the current intensity incrementally increased. If contraction occurred, the corresponding current intensity was recorded as the stimulation threshold under deep NMB.

After all measurements were completed, GA was maintained using TIVA, and surgery was initiated under deep NMB.

### 2.5. Outcomes

The primary outcome of this study was to evaluate the minimal current intensity required to elicit ALM contraction during the progress of NMB. Finally, this study aimed to identify the corresponding stimulation threshold, which was assessed 1 min after TOFc 0, to evaluate whether ALM contraction persisted under complete NMB.

Secondary outcomes included the current intensity thresholds corresponding to each TOFr level from 90% to 10% and TOFc from 4 to 1, as well as the occurrence of intraoperative obturator reflex or the need to interrupt the procedure due to reflex-mediated adductor muscle contraction.

In addition, the following clinical variables were recorded: intraoperative events (obturator reflex, perforation, and hemorrhage) and duration of surgery and anesthesia. Tumor characteristics were obtained from operative and pathological reports, including the number of tumors, anatomical location (lateral/trigone/multiple), clinical stage, and histological grade.

### 2.6. Statistical Analysis

The sample size was estimated based on a within-subject comparison framework using a paired *t*-test model, assuming a medium effect size (Cohen’s d = 0.5) in the absence of prior clinical data. A significance level of 0.10 was selected in line with the exploratory nature of this study, with statistical power set at 80%. This yielded a minimum required sample size of 26 participants; to account for potential dropouts or missing data, we recruited 30 patients.

A control group was not included due to ethical concerns. Assigning patients to undergo TURBT near the lateral bladder wall without obturator nerve block or NMB would expose them to a high risk of obturator reflex-induced complications. Therefore, a single-arm, within-subject design was adopted to minimize patient risk while still allowing meaningful observational analysis.

Statistical analysis was performed using IBM SPSS Statistics for Windows, version 28.0 (IBM Corp., Armonk, NY, USA). The distribution of continuous variables was assessed using both the Kolmogorov–Smirnov and Shapiro–Wilk tests. Age, weight, BMI, duration of surgery, and duration of anesthesia followed a normal distribution and were expressed as the mean ± standard deviation (SD). Height, which did not satisfy the normality assumption, was reported as the median with the interquartile range (IQR). Categorical variables, including sex, American Society of Anesthesiologists physical status, tumor characteristics (number, location, stage, and grade), and intraoperative events (obturator reflex, perforation, and hemorrhage), were summarized as counts and percentages. Given the single-arm, observational design of this study, no inter-group hypothesis testing was performed. All statistical analyses were descriptive in nature.

Descriptive statistics were used to summarize the current intensity values required to elicit ALM contraction at each stage of neuromuscular blockade. Stages were categorized sequentially from TOFr 90% to 10%, followed by TOFc 3 to 0. Current intensity values were reported as the mean ± SD, median, IQR, and range for each stage. Current intensity values at TOFc 0 were analyzed separately to assess the persistence of the obturator reflex under complete NMB.

Additionally, individual-level changes were explored using a linear mixed-effects model (LMM) to account for within-subject variability. To evaluate the overall trend in current intensity while controlling for within-subject correlations and baseline variability among patients, the TOF stage was treated as a fixed effect, and patient-specific differences were modeled using a random intercept.

In addition, a post hoc multivariable linear regression analysis was performed to evaluate whether baseline clinical variables—including age, sex, BMI, ASA physical status, tumor number, location, stage, and grade—were associated with the stimulation threshold required to elicit adductor longus muscle contraction at TOFc 0.

## 3. Results

A total of 30 patients scheduled to undergo TURBT under GA were screened and found to meet all eligibility criteria. Written informed consent was obtained from each participant before enrollment. All 30 patients completed the study protocol and were included in the final analysis without any exclusions. The demographic and clinical characteristics of the study population are summarized in Table 1.

**Figure 2:**
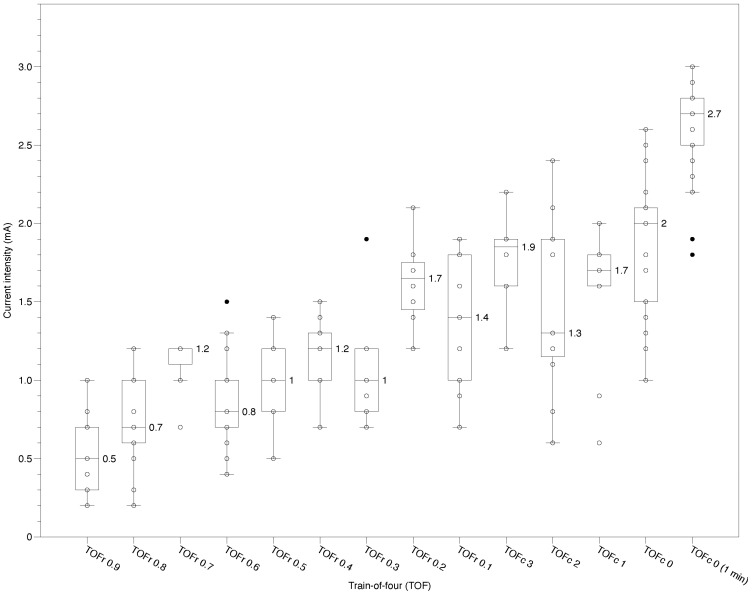
Box-and-scatter plot of the minimal current intensity required to elicit contraction of the adductor longus muscle in response to obturator nerve stimulation at each stage of neuromuscular blockade. The TOF stages are arranged in sequential order from TOFr 90% to TOFc 0, including TOFc 0 (1 min), which represents a measurement taken 1 min after TOFc 0. Each circle represents an individual measurement of current intensity corresponding to a specific TOF count or TOF ratio. Black dots represent outliers. The box plot shows each TOF stage’s median, interquartile range (IQR), and spread of the current intensity values. TOFr = Train-of-Four ratio; TOFc = Train-of-Four count.

As NMB deepened, the current intensity required to elicit ALM contraction progressively increased (Table 2 and Figure 2). At TOFr 90%, the mean intensity was 0.51 ± 0.25 mA, rising to 1.66 ± 0.53 mA at TOFr 10%. During the TOFc stages, it further increased from 1.68 ± 0.59 mA at TOFc 3 to 2.04 ± 0.66 mA at TOFc 0. Under conditions of complete NMB, which was assessed 1 min after TOFc 0, all 30 patients exhibited the ALM contraction in response to electrical stimulation of the obturator nerve, indicating the persistence of the obturator reflex despite deep NMB. At this time point, the required current intensity was 2.61 ± 0.29 mA, with a median of 2.70 mA and an interquartile range of 2.50–2.80 mA.

An LMM was applied to analyze the increasing trend in stimulation thresholds across progressive NMB while accounting for repeated measurements with missing data at specific NMB stages. The model included the TOF stage index as a fixed effect, and patient-specific variation was modeled using random intercepts. The analysis revealed that the fixed effect of the stage was statistically significant (β = 0.133, *p* < 0.001), indicating that the current intensity required to provoke ALM contraction consistently increased across TOF stages. This result supports a robust association between deepening NMB and elevated current intensity requirements while accounting for within-subject correlation.

A multivariable linear regression analysis was conducted to assess the association between patient characteristics (age, BMI, ASA physical status, and tumor-related factors) and the current threshold required to elicit adductor muscle contraction at TOFc 0 (Table 3). However, none of the variables showed a statistically significant association (all *p* > 0.5), and the overall model fit was poor (R^2^ = 0.022, *p* = 0.965), suggesting that these clinical factors may not meaningfully influence the stimulation threshold under deep NMB in this study.

## 4. Discussion

In this study, we observed that all patients (100%) exhibited ALM contraction in response to electrical stimulation of the obturator nerve, even 1 min after reaching complete NMB. At this point, the mean current intensity required to elicit contraction was 2.61 ± 0.29 mA, suggesting that the obturator reflex may persist despite deep NMB. The LMM analysis demonstrated that graded elevation in threshold current was statistically significant (*p* < 0.01). This reinforces the potential need for ONB even under GA.

During TURBT on the lateral wall, the obturator reflex may occur in 20–55% of patients, largely due to the close anatomical relationship between the obturator nerve and the lateral pelvic structures [9]. Although the obturator reflex is generally considered preventable with the administration of sufficient NMBAs under GA, several clinical and experimental studies have shown that this reflex may still occur even under deep NMB [6,7,10]. Soberón et al. [10] reported that the obturator reflex still occurred in approximately 21% of patients under NMB, versus only ~4% of those who received an ONB. Notably, in their study, the NMBA was administered as a single bolus during induction, which likely resulted in a progressively shallower NMB over time. This insufficient depth of blockade may have contributed to the occurrence of the obturator reflex despite the use of NMB. Koo et al. [6] demonstrated that deep NMB significantly reduces the incidence of obturator reflex and improves surgical conditions, but their findings also indicate that the obturator reflex may not be completely abolished even under deep NMB, which is a noteworthy observation. The observation that adductor muscle contractions can be elicited during TURBT, despite the administration of NMBAs, raised the need to verify whether such reflex responses are truly possible under deep NMB. Furthermore, without clear guidelines, it was uncertain whether ONB should be routinely performed. In light of these unresolved issues, we conducted this exploratory study to evaluate whether ALM contraction persists under deep NMB and to determine the electrical threshold required to elicit such reflexes.

Unlike common spinal reflexes, the obturator reflex is believed to be elicited by the direct electrical stimulation of the obturator nerve from energy delivered during surgical procedures. Anatomically, the obturator nerve originates from the lumbar plexus (L2–L4), descends along the pelvic wall, and passes close to the inferolateral bladder wall before exiting through the obturator canal to innervate the medial thigh [11]. Bladder wall thickness (BWT) in adults is generally around 3–5 mm [12]. However, BWT varies depending on the degree of bladder filling: it becomes thicker when the bladder is nearly empty and thins as it becomes fully distended [13]. During TURBT, especially when the bladder is filled with irrigation fluid and overdistended, the lateral wall of the bladder is positioned adjacent to the obturator nerve, increasing the likelihood of direct nerve stimulation during tumor resection involving the lateral wall [14]. The electrical current transmitted in such cases can activate motor fibers of the obturator nerve, resulting in a simultaneous and forceful contraction of multiple adductor muscle groups.

Theoretically, NMBAs completely abolish nerve-to-muscle transmission by binding to acetylcholine (ACh) receptors at the neuromuscular junction (NMJ), which could prevent the obturator reflex under NMB. However, even a deep block on the neuromuscular transmission (NMT) monitoring is rarely 100%—a fraction of ACh receptors may remain unoccupied [15]. This study measured the final current intensity one minute after achieving a TOFc of 0. However, it is important to recognize that although a TOFc of 0 indicates deep NMB, a temporal gap remains before achieving complete blockade as defined by a post-tetanic count (PTC) of 0 [8]. Moreover, the degree of NMB may differ between muscles, depending on their vascularity and sensitivity to neuromuscular blocking agents [16,17]. Such differences can lead to discrepancies in NMT monitoring at the adductor pollicis muscle and the actual blockade status of the thigh adductor muscles, potentially allowing the obturator reflex to occur even when monitoring suggests deep blockade. Likewise, differences in recovery speed due to variations in blood supply and receptor sensitivity among different muscles can result in a further mismatch between monitoring data and clinical observations. In addition, without continuous administration of NMBAs, the obturator reflex—although initially suppressed by adequate NMB after the induction of GA—may reappear during surgery as the progressive metabolism and redistribution of NMBAs proceed. Notably, such recurrence may occur despite deep NMB, reflecting the limitations of NMT in its ability to precisely predict the occurrence of the obturator reflex during TURBT [6]. Therefore, prophylactic ONB may be a reasonable adjunct to prevent the obturator reflex during GA.

In addition to these clinical and pharmacologic considerations, the neurophysiological mechanism of post-tetanic facilitation may also contribute to the reappearance of the obturator reflex under neuromuscular blockade. Strong or repetitive neural stimulation can cause the motor nerve endings to release a surge of ACh, which may transiently overcome the blockade at those remaining receptors, which is known as post-tetanic facilitation [18]. Therefore, even under deep NMB, strong stimulation can mobilize hidden ACh stores, allowing a transient muscle response. During TURBT, electrocautery often delivers rapid electrical impulses similar to tetanic stimulation. This may induce a surge of ACh at the synapse, temporarily overcoming NMB at certain receptors and eliciting partial muscle activation. Consequently, intense obturator nerve stimulation can still provoke adductor spasms, accounting for the incomplete suppression of the obturator reflex despite NMBA administration. Soberón et al. demonstrated that the obturator reflex still occurred in patients under NMB conditions with a TOFc 0 or 1, indicating that even an absence of TOF twitches may not be sufficient to prevent adductor spasm during TURBT [10]. Consequently, it is presumed that a substantial ACh surge would exert little to no clinical effect only under conditions approaching complete NMB (PTC < 1), as the limited availability of postsynaptic receptors during complete blockade makes muscle contraction virtually impossible.

In an exploratory post hoc analysis, we conducted a multivariable linear regression to assess whether any patient or tumor-related factors—such as age, BMI, tumor number, location, stage, or grade—could predict the stimulation threshold required to elicit an adductor contraction under deep NMB. However, no statistically significant associations were found. Since the stimulus was applied directly to the nerve rather than being transmitted through surrounding tissues, factors like tumor location or stage would not meaningfully alter the reflex threshold. Similarly, patient demographics such as sex, BMI, or age are more likely to affect the pharmacodynamics of neuromuscular blockade, such as its onset time or duration, rather than the electrophysiological response of the nerve itself [19,20]. Therefore, it may be reasonable to assume that the current intensity required to provoke the obturator reflex in this model primarily reflects the level of neuromuscular blockade achieved at the time of stimulation, rather than any anatomical or demographic variable. This observation suggests that achieving and maintaining a profound level of blockade—possibly beyond TOFc 0—might be necessary to suppress obturator nerve-mediated responses during TURBT.

The persistence of adductor muscle contractions at TOFc 0 has important implications for intraoperative safety. It suggests that anesthesiologists and urologists should not assume that a patient under full muscle relaxant paralysis is free from the obturator reflex. These findings may suggest that, in clinical settings involving bladder tumors near the lateral wall, employing an obturator nerve block or other preventive strategies could still be warranted, even when a deep neuromuscular blockade is applied. Our data support the idea that ONB remains a valuable adjunct to prevent obturator reflex during TURBT [10]. Multiple studies and a recent meta-analysis have confirmed that adding an ONB significantly reduces the incidence of obturator jerks and associated complications during TURBT [21]. Therefore, while deep NMB can reduce the risk of reflexive adductor contractions, the addition of an ONB may offer further protective benefit in lateral wall tumors and could be considered as part of a multimodal strategy to minimize intraoperative complications.

### Limitations

This study has several limitations. First, it employed a single-arm design without a comparator group, limiting the generalizability of the findings. The absence of a control group—such as a cohort receiving GA without ONB—was a methodological constraint primarily driven by ethical concerns. Conducting TURBT without ONB in patients with tumors near the lateral bladder wall would pose a significant risk for obturator reflex-related complications, including bladder perforation, and was therefore not feasible in our institution. Second, due to the nature of NMT monitoring, which was measured at 12 s intervals, missing data at specific stages was inevitable. To mitigate this issue, an LMM was used to account for within-subject variability and estimate effects across varying depths of neuromuscular block, although this approach cannot fully replace structured sampling. Third, we performed the final measurement one minute after observing a TOFc of 0, assuming that a sufficiently deep NMB had been achieved. However, as a complete blockade defined by a PTC of 0 may require an additional 1 to 2 min, it is possible that full receptor occupancy had not yet been reached in some patients. Therefore, if the final measurement had been performed at a later time point, the results might have differed. Continuous intraoperative monitoring could also have provided more definitive data regarding the progression of neuromuscular blockade. However, due to the practical conditions of performing surgery, such an approach was not feasible in the clinical setting. Lastly, the electrical properties of pelvic tissues were estimated using theoretical modeling. However, based on published dielectric data, these approximations may not fully capture pelvic structures’ anatomical and electrical complexity. Additionally, the use of a significance threshold of 0.10 for sample size estimation reflects the exploratory nature of this study. As such, the findings should be interpreted as hypothesis-generating rather than definitive, warranting validation in future controlled trials.

## 5. Conclusions

This study’s findings that ALM contractions in response to obturator nerve stimulation persisted in all patients during TURBT despite achieving deep NMB (TOFc 0) raise concerns that deep NMB alone may be insufficient to prevent obturator reflex and suggest that ONB should be considered as an adjunctive practice during TURBT under GA in patients at risk. However, given the single-arm design and absence of a comparator group, these results should be interpreted cautiously. The results should be viewed as hypothesis-generating, and further randomized controlled studies are needed to clarify the relative contributions of NMB and ONB and to establish evidence-based protocols for reflex prevention during TURBT.

## Figures and Tables

**Table 1:** Patient’s characteristics.

Factors	(*n* = 30)
Age (yr)	72.5 ± 8.5
Sex (male/female)	28 (90.3)/2 (6.5)
Height (cm)	166.5 (164.1–170.0)
Weight (kg)	65.4 ± 10.3
BMI (Kg/m^2^)	23.9 ± 2.6
ASA-PS (I/II)	12 (38.7)/19 (12.9)
Tumor stage	
Tis/Ta/T1/T2	3 (9.7)/18 (58.1)/5 (16.1)/5 (16.1)
Tumor grade	
No cancer/Low/High	5 (19.3)/9 (29.0)/16 (51.6)
Tumor multiplicity	
Single/Multiple	11(35.5)/20 (64.5)
Tumor location	
Lateral/Trigone/Multiple	9 (29.0)/4 (12.9)/18 (58.1)
Duration of surgery (min)	48.3 ± 12.7
Duration of anesthesia (min)	74.5 ± 14.1

Values are expressed as the mean ± standard deviation (SD) for normally distributed continuous variables and median with interquartile range (IQR) for non-normally distributed variables, as determined by the Shapiro–Wilk test. Categorical variables are presented as a number (%). BMI = body mass index; ASA-PS = American Society of Anesthesiologists physical status; Tis = Carcinoma in situ; Ta = Non-invasive papillary carcinoma.

**Table 2:** Summary of current intensity required to elicit ALM contraction across TOF stages.

TOF Stage		Number of Observations	Mean ± SD	Median (IQR)
TOF ratio	0.9	25	0.51 ± 0.25	0.50 (0.30–0.70)
	0.8	22	0.73 ± 0.26	0.70 (0.60–0.95)
	0.7	7	1.10 ± 0.19	1.20 (1.10–1.20)
	0.6	15	0.89 ± 0.30	0.80 (0.70–1.00)
	0.5	5	0.98 ± 0.35	1.00 (0.80–1.20)
	0.4	10	1.17 ± 0.23	1.20 (1.05–1.27)
	0.3	9	1.07 ± 0.37	1.00 (0.80–1.20)
	0.2	8	1.62 ± 0.27	1.65 (1.48–1.73)
	0.1	11	1.37 ± 0.43	1.40 (1.00–1.80)
TOF count	3	6	1.77 ± 0.34	1.85 (1.65–1.90)
	2	12	1.47 ± 0.54	1.30 (1.18–1.90)
	1	9	1.54 ± 0.48	1.70 (1.60–1.80)
	0	30	1.87 ± 0.44	2.00 (1.50–2.08)
A minute after the TOF count 0		30	2.61 ± 0.29	2.70 (2.50–2.80)

Current intensity values represent the minimal electrical stimulation (in mA) required to elicit contraction of the adductor longus muscle (ALM) in response to obturator nerve stimulation at various stages of Train-of-Four (TOF) monitoring. Data are expressed as the mean ± standard deviation (SD) and median with interquartile range (IQR, 25th–75th percentile).

**Table 3:** Multivariable linear regression analysis of factors associated with stimulation threshold at TOFc 0.

Variable	β Coefficient	Standard Error	*p*-Value
Age	0.015	0.038	0.71
BMI	−0.042	0.097	0.67
Sex (male)	0.121	0.213	0.58
ASA-PS (II vs. I)	−0.134	0.189	0.49
Tumor number	0.038	0.079	0.63
Lateral location	0.089	0.151	0.56
Stage (T1 vs. Ta)	0.071	0.128	0.58
Grade (High vs. Low)	0.053	0.146	0.72

Multivariable linear regression was performed to assess the association between baseline patient/tumor characteristics and the stimulation threshold required to elicit adductor longus muscle contraction at TOFc 0. None of the variables were statistically significant (all *p* > 0.05). TOFc = Train-of-Four count; BMI = body mass index; ASA = American Society of Anesthesiologists physical status classification; β = regression coefficient.

## Data Availability

The data presented in this study are available upon request from the corresponding author because IRB approval did not include provisions for sharing raw data externally.

## Abbreviations

The following abbreviations are used in this manuscript:

ACh	Acetylcholine
ALM	Adductor Longus Muscle
ASA	American Society of Anesthesiologists
BMI	Body Mass Index
GA	General Anesthesia
LMM	Linear Mixed Model
NMB	Neuromuscular Blockade
NMBA	Neuromuscular Blocking Agent
NMT	Neuromuscular Transmission
ONB	Obturator Nerve Block
PTC	Post-Tetanic Count
TOF	Train-of-Four
TOFc	Train-of-Four Count
TOFr	Train-of-Four Ratio
TURBT	Transurethral Resection of Bladder Tumor
TIVA	Total Intravenous Anesthesia
